# A case report of membrane induction combined with RIA technique for the repair of distal humerus segmentary bone defect

**DOI:** 10.3389/fendo.2023.1150029

**Published:** 2023-08-21

**Authors:** Guoliang Wang, Zhenfeng Zhu, Shuaikun Lu, Linhu Wang, Hao Gao, Congxiao Fu, Jun Ren, Xiang Liu, Yong Zhang, Yunfei Zhang

**Affiliations:** Department of Orthopaedics, Tangdu Hospital, Air Force Medical University, Xi’an, China

**Keywords:** bone defect, distal humerus, membrane induction technology, repair and reconstruction, RIA (Reamer-Irrigator-Aspirator)

## Abstract

Bone nonunion and bone defect are common postoperative complications in clinic. Membrane induction or Ilizarov technique is often used to repair bone defect. Autologous bone is often used for bone defect repair and reconstruction, and the anterior superior iliac spine, posterior superior iliac spine or fibula bone is used as the donor area for bone extraction, but there are problems of donor area complications. In recent years, the development of bone marrow aspiration (RIA) has provided a new alternative way for the source of autogenous bone. We report a 48-year-old female patient with a comminuted supracondylar intercondylar fracture of the left humerus due to a car accident. After 8 months of emergency debridement and suture with Kirschner wire internal fixation, the fracture was found to be unhealed with extensive bone defects. We used membrane induction combined with RIA technology to repair and reconstruct the patients, and found good osteogenesis through late follow-up. In theory, membrane induction technique can realize the reconstruction of large segmental bone defects, but the scope of repair is often limited by the lack of autologous bone source. The emergence and development of RIA technology provides us with a new autologous bone donor area for bone repair and reconstruction surgery. It can provide a large amount of high-quality cancellar bone mud through minimally invasive means. Meanwhile, it can reduce patients’ pain, infection, fracture, aesthetics and other problems caused by iliac bone extraction, and shorten patients’ bed time. Maximize the preservation of the patient’s autologous bone source. For the first time in the world, we reported the combination of membrane induction technology and RIA technology in the treatment of segmental bone defects, providing a new idea for the treatment of bone defects.

## 1 Introduction

Bone nonunion and bone defect are common postoperative complications of fracture in clinical practice, and their causes are usually related to unstable fracture ends, poor osteogenic ability, infection and other factors. For nonunion without obvious defect, primary bone graft can be used for repair. However, for nonunion with large defect, primary bone graft may cause poor bone resorption and osteogenesis, with poor effect. Therefore, membrane induction technique or Ilizarov technique is often used for repair and reconstruction. In particular, membrane induction technique requires the use of a large amount of autologous bone in the secondary repair and reconstruction. These autologous bones are often obtained from the anterior superior iliac spine, posterior superior iliac spine or fibula, but high-quality cancellous bone is limited. If more high-quality autogenous bone can be obtained, larger bone defects can be repaired by membrane induction technique. In recent years, the development of bone marrow aspiration (RIA provides the full English name of RIA) technology has provided a new option for the source of autologous bone (1–3). This technology can be used to minimally extract more autologous bone mud rich in stem cells from patients’ tubular bone, with good bone condition and strong osteogenic ability (4). We report here for the first time a membrane-induced RIA technique for the repair of a 4cm segmentary bone defect in the distal humerus.

## 2 Case presentation

**Figure 1:**
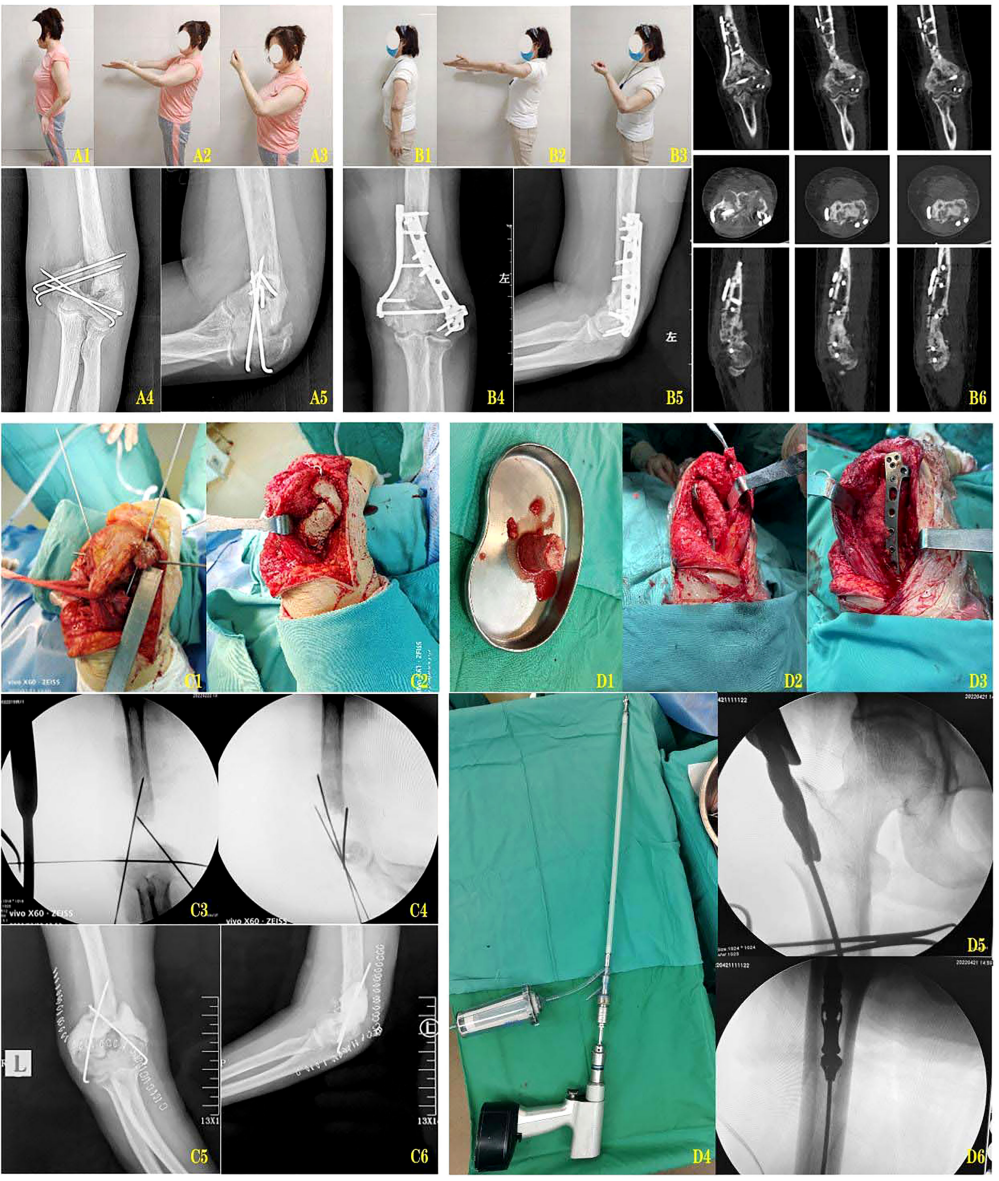
**(A1-A3)** The posterior function of the fixation of the fracture. **(A4-A5)** The left elbow of the posterior left elbow and the lateral X-ray. **(B1-B3)** 10 months after Masquelet induction and RIA bone graft reconstruction. **(B4, B5)** Posterior lateral X-rays of the left elbow joint at 10 months after internal fixation reconstruction. **(B6)** CT scan at 10 months after internal fixation reconstruction. **(C1, C2)** The membrane induced phase of the process. **(C3, C4)** The image of the membrane induced phase. **(C5, C6)** The membrane is induced by the left elbow and the lateral position X-ray. **(D1)** The pine bone clay removed from the myotor. **(D2, D3)** Use the medullary bone graft bone to rebuild the distal humerus. **(D4)** The use of the RIA reaming device is widely used. **(D5, D6)** RIA was expanded with the image of the bone.

A 48-year-old female patient came to our outpatient clinic for nonunion after internal fixation for comminuted supracondylar fracture of the left humerus intercondylar. She suffered from an open supracondylar comminuted fracture of the left intercondylar humerus caused by a car accident eight months ago. She was given defibrillated suture and Kirschner wires internal fixation in the local hospital in the emergency department. During the postoperative review, the fracture was found to be unhealed, with loose internal fixation failure and displacement of the broken end into angular deformities. At presentation, there was significant absorption of the broken end of the fracture, and the patient complained of pain and limited mobility of the left elbow. Physical examination revealed enlargement and deformity of the left distal humerus, normal skin temperature, no abnormal skin color, obvious tenderness of the distal humerus, palpable slight bone rubbing sensation, elbow flexion and extension motion of about 20°-90°, forearm rotation dysfunction, peripheral blood flow and good feeling. Laboratory examination indicated that WBC, ESR and C-reactive protein indexes were all within the normal range. Imaging examination showed that the fracture was not healed and the broken end of the fracture was clearly angled. After preoperative preparation was improved and no abnormalities were found, intraoperative exploration revealed a 4cm bone defect in the distal humerus, and a membrane-induced first-stage operation, namely debridement exploration and internal fixation, fracture reduction and internal fixation, was arranged for the patient. After an interval of 8 weeks, laboratory indicators were reviewed and combined with gross symptoms to rule out infection. In the second stage, the bone cement was removed by open exploration, and the left femoral bone marrow was removed by RIA technology. During the operation, RIA technology was used to extract about 40ml autologous bone mud from the patient’s left femoral bone marrow cavity to repair the 4cm bone defect of the distal humerus. Postoperative follow-up was carried out, and X-ray and CT examinations were reviewed at 6 weeks, 3 months, 4 months, 5 months and 6 months, respectively. The results showed good healing of the bone graft area and no loosening of internal fixation. In the reexamination 6 months after surgery, the function of the left elbow was significantly improved, the elbow motion was about 0°-120°, and the forearm pronation and supination were good (**Figure 1**).

## 3 Discussion

At present, membrane induction, bone removal and bone graft with vascular pedicle are usually used to repair a large range of bone defects. However, membrane induction seems to be more dominant for the bone defects around the joint. A large number of autogenous bones were used in the membrane-induced secondary surgery, usually from the anterior superior iliac spine and posterior superior iliac spine. However, cancellous bone grains were mostly used, and their volume was limited, especially for patients with autogenous iliac bone, and bone size, plasticity degree and filling gap were uneven. Bone mud extracted from the diaphyseal medullary cavity by RIA technology is rich in a large number of stem cells and rich in growth factors (5), with better filling effect and greater plasticity, which is especially suitable for the repair of irregular metaphysis defects. Besides, the contact area of bone mud is larger than that of bone grains and bone blocks, and the osteogenesis ability is stronger, and the callus quality is higher (6). At the same time, the risk of pulmonary embolism has been significantly reduced by current RIA technology (7–11). However, RIA bone extraction also has certain limitations. RIA is usually used to extract bone from femoral bone marrow. If the femur is diseased or the femoral bone marrow cavity is too thick, bone extraction may not be possible or affect the amount of bone extraction. There may also be complications such as cortical perforation, blood loss and infection (12, 13).

In this case membrane induction and RIA were used for the first time to repair segmentary bone defects in the distal humerus. Imaging review at 6 weeks after bone graft reconstruction showed blurred bone graft area, good plasticity in the distal supracondylar area of the humerus, no abnormal osteogenesis in the joint space, and more importantly, no complications such as myositis ossificans. The patient underwent early flexion and extension exercises and achieved good elbow function. Dynamic follow-up was conducted from 3 months to 6 months after the operation, and good osteogenesis was observed in the bone graft area. Compared with bone grain grafting, the use of bone mud bone grafting has better plasticity, higher osteogenic quality and less impact on flexion and extension of the elbow.

Membrane induction technology provides a good osteogenic environment through biofilm and avoids bone graft absorption. RIA technology can provide more high-quality autogenous bone on the basis of the original bone source. The combination of the two technologies can provide a new possibility for the reconstruction of larger segments of bone defect.

## Data availability statement

The raw data supporting the conclusions of this article will be made available by the authors, without undue reservation.

## Ethics statement

The studies involving human participants were reviewed and approved by Medical Ethics Committee of the Second affiliated hospital, Air Force Medical University. Written informed consent was obtained from the participant/patient(s) for the publication of this case report.

## Author contributions

GW contributed to data collection and writing the paper and performed surgeries. ZZ and SL contributed to data collection and data analysis, performed surgeries, and wrote the paper. LW and HG contributed to data collection. CF, JR and XL contributed to patient follow-up. YuZ and YoZ contributed to overall planning and data analysis and performed surgeries. YuZ is the first corresponding author. GW, ZZ and SL share the first authorship. All authors contributed to the article and approved the submitted version.
